# Impact of Climate Change on Phenology of Two Heat-Resistant Wheat Varieties and Future Adaptations

**DOI:** 10.3390/plants11091180

**Published:** 2022-04-27

**Authors:** Muhammad Ishtiaq, Mehwish Maqbool, Mahnoor Muzamil, Ryan Casini, Abed Alataway, Ahmed Z. Dewidar, Ahmed M. El-Sabrout, Hosam O. Elansary

**Affiliations:** 1 Department of Botany, Mirpur University of Science and Technology, Mirpur-10250 (AJK), Pakistan; mehwish.botany@must.edu.pk (M.M.); mahnurhussain10@gmail.com (M.M.); 2 School of Public Health, University of California, 2121 Berkeley Way, Berkeley, CA 94704, USA; ryan.casini@berkeley.edu; 3 Prince Sultan Bin Abdulaziz International Prize for Water Chair, Prince Sultan Institute for Environmental, Water and Desert Research, King Saud University, Riyadh 11451, Saudi Arabia; aalataway@ksu.edu.sa (A.A.); adewidar@ksu.edu.sa (A.Z.D.); 4 Agricultural Engineering Department, College of Food and Agriculture Sciences, King Saud University, Riyadh 11451, Saudi Arabia; 5 Department of Applied Entomology and Zoology, Faculty of Agriculture (EL-Shatby), Alexandria University, Alexandria 21545, Egypt; elsabroutahmed@alexu.edu.eg; 6 Plant Production Department, College of Food and Agriculture Sciences, King Saud University, Riyadh 11451, Saudi Arabia

**Keywords:** climate change, phenology changes, heat-resistant varieties, with Global climate models (GCM), Representative Concentration Pathway (RCP), late mature variety

## Abstract

Climate change (CC) is a global threat to the agricultural system. Changing climatic conditions are causing variations in temperature range, rainfall timing, humidity percentage, soil structure, and composition of gases in environment. All these factors have a great influence on the phenological events in plants’ life cycle. Alternation in phenological events, especially in crops, leads to either lower yield or crop failure. In light of respective statement, the present study is designed to evaluate the climatic impacts on two heat-resistant wheat varieties (Sialkot–2008 and Punjab–2018). During the study, impacts of CC on wheat phenology and annual yield were predicted considering six climatic factors: maximum temp, minimum temperature, precipitation, humidity, soil moisture content, and solar radiation using two quantitative approaches. First, a two-year field experimental plot was set up at five different sites of study—each plot a bisect of two sites. Phenological changes of both varieties were monitored with respect to climatic factors and changes were recorded in a scientific manner. Secondly, experimental results were compared with Global climate models (GMC) models with a baseline range of the past 40 years (1970–2010) and future fifty years (2019–2068) under Representative Concentration Pathway (RCP) 8.5 model analysis. Field experiment showed a (0.02) difference in maximum temperature, (0.04) in minimum temperature, (0.17) in humidity, and about (0.03) significant difference in soil moisture content during 2019–2021. Under these changing climatic parameters, a 0.21% difference was accounted in annual yield. Furthermore, the results were supported by GMC model analysis, which was analyzed by Decision Support System for Agrotechnology Transfer (DSSAT) model. Results depicted that non-heat-resistant wheat varieties could cause up to a 6~13% reduction in yield during future 50 years (2019–2068)) compared with the last 40 years (1970–2010). A larger decline in wheat grain number relative to grain weight is a key reducer of wheat yield, under future climate change circumstances. Using heat-tolerant wheat varieties will not only assist to overcome this plethora but also provide a potential increase of up to 7% to 10% in indigenous environment. On the other hand, it was concluded that cultivating these heat-resistant varieties that are also ripening late culminates into enhanced thermal time chucks during the grain-filling period; hence, wheat yield will increase by 8% to 12%. In changing climatic conditions and varieties, ‘Punjab–2018′ will be the better choice for peasants and farm-land owners to obtain a better yield of wheat to cope with the necessities of food on the domestic and national level.

## 1. Introduction

Worldwide and regional alterations in annual temperature and precipitation patterns present a solid foundation of evidence that the global climate is changing. Climatic alterations are found to impact agricultural output widely depending on extent and geographical zone [1]. According to research evidences, IPCC predicted that a 1–3 °C increasing global temperature after 1990 would positively impact some regions while a negative impact would be observed in other parts of the world [2,3]. Positive effect of changing climate (CC) with respect to agricultural production will be permanent in areas lying at 55° north [4] while dry and hot areas will receive severe negative impacts due to changing climate [5]. Pakistan and some other developing countries lie in the most effective zone due to changing climate, i.e., rising temperature and lower/irregular rainfall [6], which increases the probabilities of intense climatic events such as (drought, heat, coldness, and flood), particularly in developing countries threatening agricultural production to extreme levels that may culminate into starvation [7]. These severe climatic events are predicted to cause food insecurity in agricultural-based developing and underdeveloped countries [8].

The second major contributor to GDP in Pakistan is the agricultural sector. In 2012–2013, sugar cane, wheat, rice, and maize contributed 6% to GDP [9]. The most important agricultural crop from a nutritional point of view is wheat; for this reason, it provides about 48% of daily calorie intake and is a so-called ‘food security pillar’ [10]. Crops play a dominant role in GDP worldwide, i.e., 10.1%, of which wheat contributes about 2.2% share [9]. Population in Pakistan is increasing day-by-day since independence while the area for crop cultivation is just 40% creating pressure on cultivation land along with climatic challenges [11].

According to FAO, around 21% of the world consumes wheat (*Triticum aestivum*) crop as a source of major nutrition, cultivated on 200 million hectares of land all over the world (http://www.fao.org) accessed on 15 April 2020. Wheat is imported and exported globally, wherein the major importers of wheat across world are developing countries (43% of food imports), with the notable fact that 81% of wheat production is observed and utilized within these countries [12]. A reducing trend in wheat production due to increasing solar radiation has been emphasized by Pathak et al. [13]. At 17–17.7 °C, terminal spikelet forms in wheat, and evidences support that an average increase of 1 °C in temperature during spikelet formation has caused a 600–650 g/m decline in annual wheat [14,15,16]. Whereas, during winters, a 0.5 °C rise in temperature could cause a 0.45 ton/ha loss in wheat annual production due to extended growth period, as wheat requires high temperature during sowing and germination but, at threshold limits, a heavy loss in yield is observable. For example, a temperature zone of 34 to 20 °C is required during September; any diversions from this temperature could have unenviable effects on establishment of seedlings, speedy/immature vegetative development, reduced canopy cover, tilling, spike bulk, and yield. Further, a temperature range of 25–10 °C during February, 30/13 °C during March, and 30/20 °C during April shrinks the number of feasible florets as does grain filling [15,17,18], and wheat production is observed at 425 ppm CO_2_ concentration without influence of climate change in both irrigated and rainfed areas [19,20].

## 2. Results

### 2.1. Field Analysis

Monthly temperature (maximum and minimum), precipitation, humidity, and solar radiation data were incorporated from nearby meteorological station during experimental trial from five selected sites (Chamb, Dander, Sarla, Kalash, and Manana). Although there was little difference between elevations of three tehsils of District Bhimber, there was protuberant visible variation observed in selected climatic parameters due to seasonal changes (Table 1).

With reference to data collected from experimental plots; it was evaluated that mean temperature (max and min), humidity, and precipitation are changing continuously according to seasonal changes. The data presented were compared to the simulated results. A significant difference of *p* = 0.05 was set to explore the difference between selected climatic parameters during two consecutive years, i.e., 2019–2021. According to simulated results, about (0.02) difference was noticed for annual maximum temperature, with a similar about (0.04) in minimum temperature, (0.17) for humidity, and about (0.03) significant difference for soil moisture content. It was noticed that the minimum temperature range showed greater significant value of difference compared with maximum temperature. No significant change was observed for solar radiation from the study area during experimental time duration.

### 2.2. Impact of Changing Climatic Parameters on Wheat Phenology

As described above, wheat phenology is highly dependent upon climatic conditions; even slight changes in atmospheric condition could cause greater changes in timing of phenological changes. For evaluating climatic impact on wheat plants development and flourishment, two wheat varieties were selected: (i) heat-resistant wheat variety Punjab–2018; (ii) less heat-resistant wheat varieties Sialkot–2008 (disease-resistant variety). Both these varieties were plotted in five experimental areas of District Bhimber, keeping soil consistency for structure and texture with the same amount of fertilizer, tillage, and watering. During experimental trials, temperature, precipitation, humidity, soil moisture content level, and solar radiation were observed per month. Phenological change of both varieties were noticed at each stage: sowing dates, first flag leave formation time, spikelet’s formation, maturity time, and yield. At the end of experimental time duration and once wheat grain were matured, all data of climatic conditions and phenological changes were compiled and analyzed. Each variety showed significant differences in phenological events attributed to changing climate in the respective areas. Table 2 describes the varying timings of the occurrence of phenological events on wheat variety.

In light of observed changes in climatic parameters, phenological events of both wheat varieties were carefully observed and noted. Both wheat varieties showed changing time duration with respect to occurrence of phenological events. It can be concluded that climate change influence on wheat phenology correlates to the cultivar used. The first level of difference was calculated for each variety, after which *t*-test was performed. For *t*-test difference, standard deviation and number of test variants were first calculated and then Pearson’s test was also performed to check degree of significance values (Table 3).

**Figure 1:**
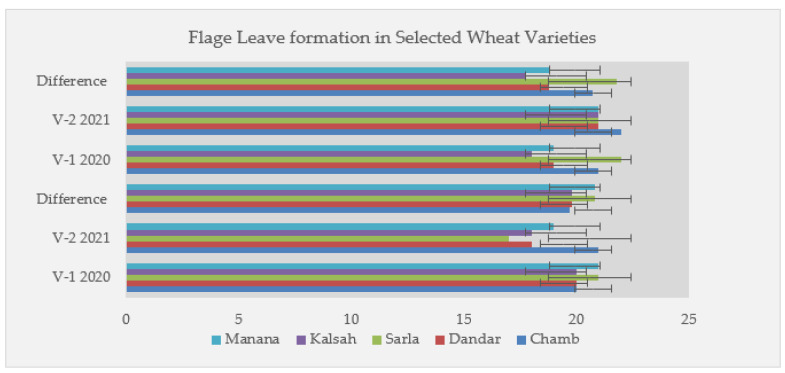

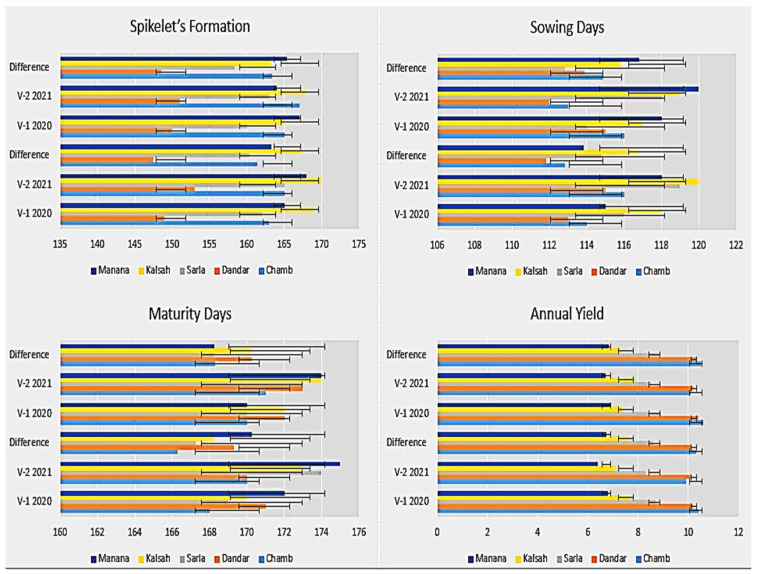
Difference between phenological events with reference to changing climatic parameters during 2019–2021 of two wheat varieties (Sialkot–2015 = V1 and Punjab–2015 = V2) from District Bhimber AJK, Pakistan.

For *t*-tests, the significance value was set to 0.05; for Pearson’s test, the significance value was set between −1 and +1 or *p* > 0.05. Results of both tests showed that all four phenological stages and annual yield of wheat (different varieties) are highly dependent on climatic parameters (temperature, precipitation, and humidity) (Table 3). However, the Punjab–2018 wheat variety showed more significant values compared with the Sialkot–2008 variety. In light of these results, we reject the H_o_; this delicts that there is a statistically significant relationship between the dependent and independent variables. So, the model is referred as an operator for prediction. Findings of Table 3 depicted that climatic parameters have a stronger influence on both wheat flourishment and developmental stages (Figure 1). Phenological time periods were compared and the significant difference level was found. According to the results, there were significant differences found between phenological time periods of wheat during two consecutive years in the same selected areas. This concludes that climatic parameters are constantly changing with shedding severe effects on the time duration of phenological events or growth cycle of wheat varieties.

To predict changing climatic pattern and support findings of experimental work, GMCs models were used. As experiments were performed for two consecutive years, and to predict climatic variation, over 30 years of data are required. So, firstly, validity of and clarification of climatic impacts on wheat flourishment were studied for two years and after assimilation of strong significant correlation evidence between climatic and phenological parameters further analysis was simulated. Two time frames were selected and analyzed, i.e., past with baseline data for forty years ranging from 1970–2010 and future (fifty years) 2019–2088 under the RCP 8.5 model. Baseline data will assist in analyzing the past trend of changing climatic parameters while future model analysis will predict the future of wheat growth and yield in coming years under changing climatic conditions.

### 2.3. Data of GCM Simulations

In present study, four GMC models—CSIRO-Mk3.6.0, GISS-E2-R, IPSL-CM5a-MR, MIROC5, and CCSM4—under RCP 8.5 scenario archived by CMIP5 were used. Detailed information on forcing data can be found on the CMIP5 website (http://cmip-pcmdi.llnl.gov/cmip5/availability.html) accessed on 15 May 2020. Many previous studies have demonstrated that the uncertainty from the CO_2_ effect is smaller than that from climate in the yield prediction, especially for C4 crop. As a consequence, only RCP 8.5, which refers to a radiative force stabilizing at 4.5 W m^−2^ by the year 2058, was selected in the study. All GCM outputs were interpolated bilinearly to a common resolution of 1.0° × 1.0° grids. The time slices of 1970–2010 and 2019–2068 were selected to represent the baseline and future climate, respectively.

### 2.4. DSSAT Model Description

DSSAT is a cropping system model successively used for analyzing growth, development, and yield production of more than 16 crop models including different wheat varieties under changing climatic parameters. DSSAT-CSM has been recognized as an effective operative tool for analyzing phenological changes observed in crop growth and yield analysis under varying growth parameters (temperature, precipitation, soil moisture content, fertilizer amount, and tillage and yield production under changing climatic conditions. Owing to workability and access to varying parameters, DSSAT-CSM was used for running GMC data.

Climatic data were incorporated from four GMC models and different analysis was simulated. Both wheat varieties’ (Sialkot–2008 and Punjab–2018) development and yield were analyzed for changing climatic factor. Simulated wheat varieties have shown responses differentially to changing temperature by firstly showing alterations in physiological processes, secondly via altering carbon assimilation rates, and thirdly by altering phenological events. Differences in phenological parameters were considered an analyzing factor and were simulated in accordance with growing degree days (GDDs). Model facilitates changes in the number of GDDs required for maturity of crop at spatiotemporal levels during long-term climatic changes. Levels of carbon in different parts of crop such as leaves, stems, and roots varies at each level of maturity and ultimately becomes zero with drying of leaves when grains are full with an increase in reproductive organs to one (i.e., 100%). To set different climatic parameters, the difference was analyzed between maximum and minimum temperature and the resultant value, i.e., 33 °C. Changing the fertilizers amount and carbon content also leads to change in assimilation. Fertilizer (NPK) was used at the seedling time once, and soil water content was determined to assume water available to roots for transpiration. All the above described changes tend to change crop yield, phenological time, and their magnitude.

**Figure 2:**
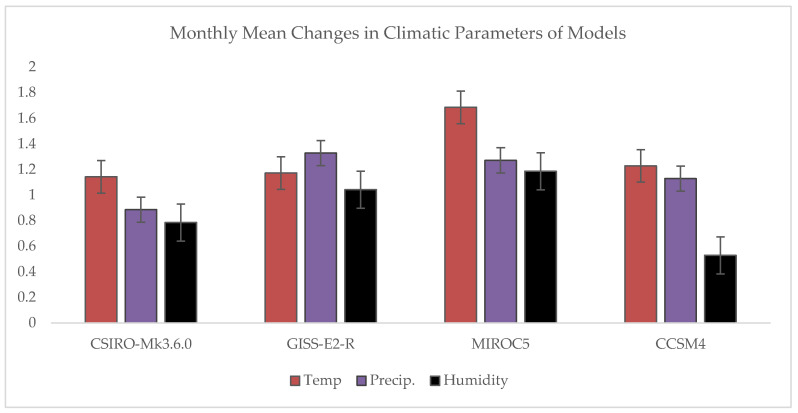
Average monthly changes in Temp (°C), precipitation (mm/day) and Humidity (%) predicted from four GMC models with Base line range of past 40 years (1970–2010) and future fifty year (2019–2068).

Comparison was made between the temperature (maximum and minimum), precipitation, and humidity values accumulated from four selected GMCs models with baseline range of forty years (1970–2010) and future range of fifty years (2019–2068). From numerical values of four GMC models, the total difference observed for average temperature was 1.41 °C, 0.88 mm/day changes in precipitation, and about 0.78% change was calculated for humidity under RCP 8.5 with respect to wheat growing season (October–April). It was noticed that temperature increased more (0.7–1.5 °C) compared to other climatic parameters (Table 4, Figure 2).

Afterwards, annual wheat production was compared with changing climatic parameters. Phenological events with reference to thermal times studied during course of study: flag leave formation (20 cd) flowering, spikelet’s formation (161 cd), and maturity (grains formation to grain filling time duration) (170 cd) at all respected sites during 2019 for the wheat variety Sialkot–2008. For Punjab–2018, flag leave formation (21 cd) flowering, spikelet’s formation (164 cd), and maturity (grains formation to grain filling time duration) (173 cd) were studied at all respective sites during 2019, with a maximum temperature above which grain number is reduced (34 °C) for both varieties. The calibrated model was then evaluated using experimental data from 2020. The coefficient of determination (*R^2^*) of the original regression lines and the root mean square errors (*RMSE*) between the simulated and measured values were calculated to evaluate model performance.

### 2.5. Simulations and Analysis

Output of four GMC models was run in DSSAT-CSM for predicting climatic changes for the future fifty years during 2019–2068, which were then compared with the corresponding baseline climatic conditions for past forty years (1970–2010). For assembling climatic effects on wheat yield under four GMC models, probability distribution was performed using two approaches: probability density functions (PDFs) and cumulative distribution functions (CDFs).

Both wheat varieties with improved heat tolerance abilities were introduced in DSSAT model. Different experimental trails were run in DSSAT: one with increased temperature, the other with increased grain-filling period, i.e., increased maturity/flowering period. For inducing higher temperature, the stressed function of the model was marked to “ON” to evaluate the wheat yield response towards higher temperature. We used the following Equation (1):

(1) $$\delta =1-0.019\left(Tmax-Tmin\right)$$

where (*δ*) represents the percentage of Grain-number fertility abridged with increasing threshold max. temperature (°C). For wheat variety Punjab–2018, limited-temperature was 34 °C. For estimating the possible effects of increased temperature on heat-resistant wheat variety, we shifted the limited temperature range to 35 °C.

In another experimental trail, the coefficient for thermal time requirements for flowering to maturity were extended, i.e., from (168 °C to 170 °C). Flowering (formation of seed on spikelet’s) to maturity of grains highly depends upon temperature, precipitation, soil moisture, and solar radiation intensity. For heat-resistant wheat variety Punjab–2018, observed thermal time duration was exceeded to evaluate impact of climate change on wheat flourishment under output of four GMC models.

### 2.6. DSSAT Model Performance

**Figure 3:**
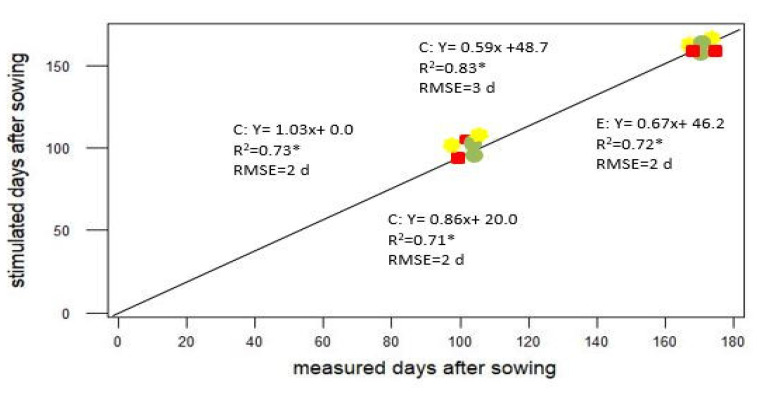

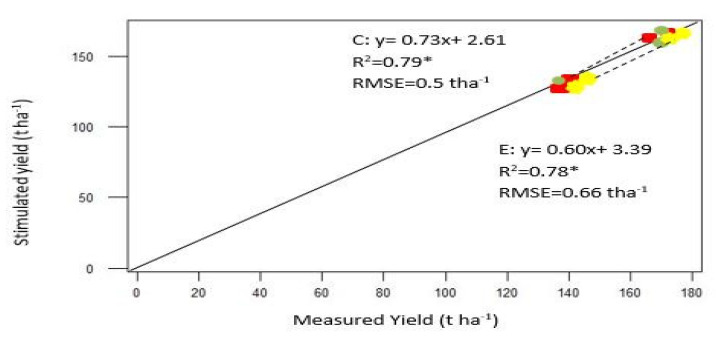
A comparative analysis showing between measured and simulated values for wheat varieties at experimental sites from District Bhimber AJK, Pakistan, for phenological events: keys: red bar (▅) indicates flowering; grey circle indicates (⏺) maturity and Yellow color asteric (⏺) yield of wheat. The asterisk (*) represent the level of the statistical significance of a regression coefficient.

Experimental trail was run for evaluating difference between values by experimental data and simulated data from GMC models by setting increased temperature. Figure 3 compares the measured and simulated values for wheat varieties at selected sites. Simulated data from GMC model were found close to observed values for flowering and maturity days. Grain yield was equitably well-anticipated. Around 72% of variation in measured phenological and yield were explained by GMC models. RMSE for simulated flowering and maturity dates were recorded after 2 and 3 days, respectively, with average error of 4% for the flowering date and 3% for the maturity date. The RMSE of simulated maize yields was 0.5–0.6 t ha^−1^, with errors generally less than 10%.

### 2.7. Climate Change Impacts

**Figure 4:**
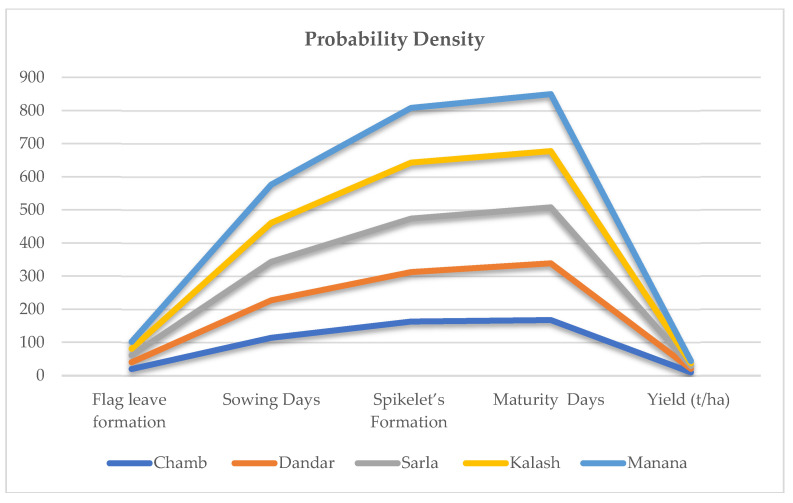

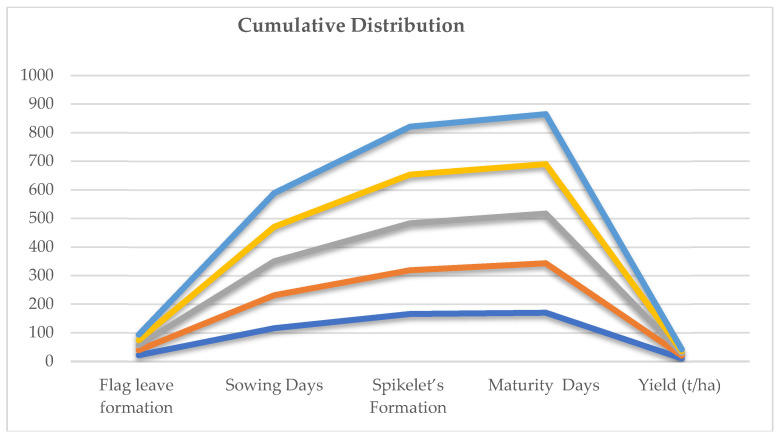
PDFs and CDFs for two wheat varieties from District Bhimber of AJK, Pakistan.

PDF and CDF changes in simulated yield observed at all selected sites are represented in Figure 4. A negative shifting of PDFs was observed for yield from both varieties and the negative tail broadens from Sarala to Manana. In general, the mean projected decreases in wheat yield ranged from 6% at Manana to 11% at Sarala. Higher probability with respect to yield changes were observed at almost all the sites with 30% to 0% interval. Conversely, probability results for changes in yield ranged from −45% to −25% and −25% to 0% intervals with approximately the same results observed at all sites. CDFs showed that the probability rate of decrease in wheat yield was more than 72%.

Changes observed in the yield are described in Figure 4. Stimulatingly, results depict that overall, the wheat grain weight is significantly increased (grains are thicker), whereas the size of grains are reportedly reduced. Projected weight of wheat grain increased at all sites from Chamb to Manana with 6 to 12%, with a probability level of 74% (Chamb) ~ 87% (Manana) during 2019–2058. Meanwhile, decrease in grain number was recorded with significantly larger percentages for historical values of 12% (Chamb) ~ 22% (Manana). CDF points feasibly decreased for wheat grain number, ranging from 84% at Chamb to 95% at Manana during 2019–2058. So, greater contribution in wheat yield was attributed to the reduction in grain number due to supraoptimal temperature shifts during flowering period.

### 2.8. Resistant Wheat Varieties

**Figure 5:**
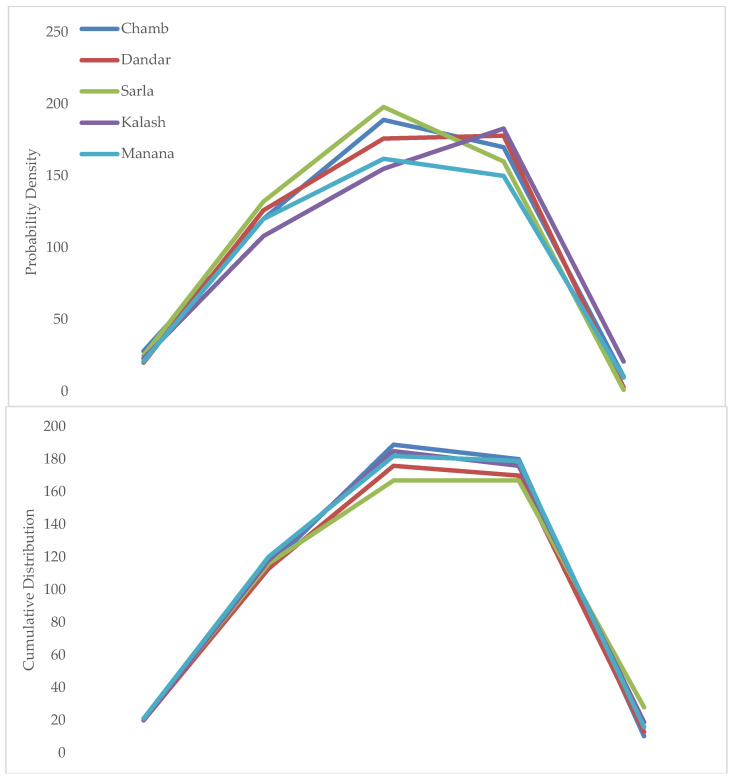
PDF and CDF values for yield changes observed for heat-tolerant wheat (Punjab–2018).

Results of PDFs showed that improved heat tolerance for grain formation of wheat varieties will show more positive results. Figure 5 shows mean of PDFs shifting more towards the positive side, which indicates relatively greater possibilities for increasing yield. The PDF comparison depicted in Figure 5 shows that yield changes are more stably dispersed. Wheat yield would increase by 7% (Chamb) ~ 10% (Kalash), with S.D. range of 13% (Chamb) ~ 18% (Kalash). Yield changes in wheat ranging from 0% to 20% interval showed maximum probability for all sites, while CDF results showed that wheat yield will increase with probability level of 67% (Chamb) ~ 75% (Kalash).

### 2.9. A Longer Grain-Filling Variety

**Figure 6:**
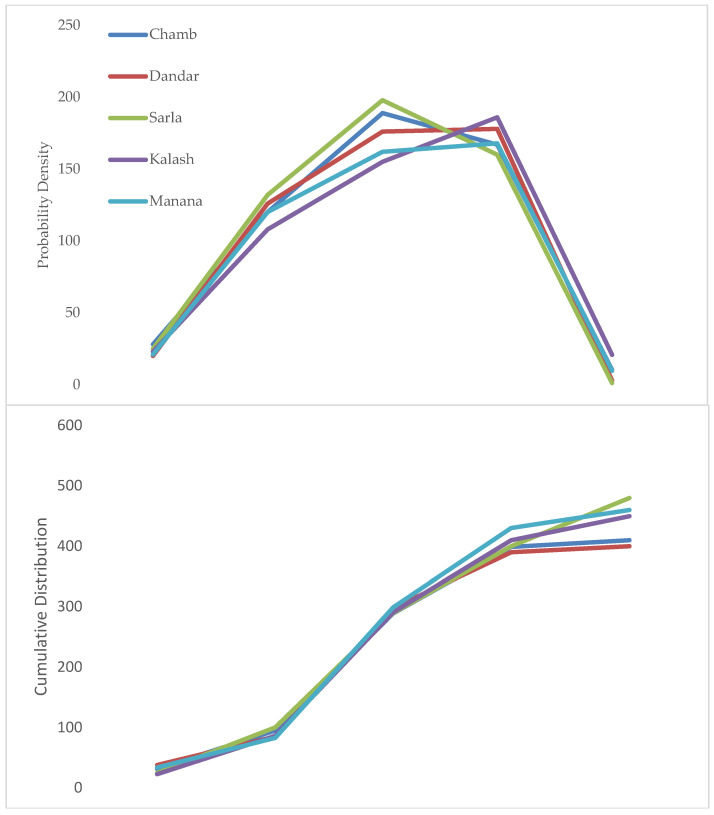
PDF and CDF values for yield changes observed for enhanced grain-filling period (Punjab–2018) variety of wheats.

Use of synthetic wheat varieties with enhanced grain-filling time duration is represented in Figure 6. Peaks of PDF show that variety has positive impacts for increasing yield, which ranges from 8% at Chamb to 12% at Kalash, while CDFs suggested that wheat yield would increase, with probability level of 68% (Sarla) ~ 78% (Manana).

## 3. Discussion

The present study provides an integrated research on assessment about the climatic impacts on wheat phenological features and its yield in District Bhimber of AJK, Pakistan. Drastic changes in climatic parameters were found to be responsible for reduced wheat yield under experimental trials and GMC model simulations confirmed the experimental work. Research study was further supported by previous studies, which depicted that changing climatic parameters are causing reductions in wheat annual yield [21,22]. Moreover, wheat grain numbers are predicted to reduce gradually under same change of climatic features in future scenarios as well. Literature show that grain number on each spikelet is highly sensitive towards changing climate. Altering climatic parameters causes changes in phenological event such as flag leave formation, spikelet’s formation, grain filling, maturity, and yield. Strong affiliation between wheat flourishment and environmental factors have been observed in both the experimental trial and simulated analysis of GCMs Barnabas et al., [23,24]. In a similar analysis, Gouache et al., exhibited that wheat varieties with enhanced thermal capacity (heat stress) during grain filling from 34 °C to 36 °C would be beneficial for offsetting the negative effects of climatic warming [25,26]. In the study using different models, it was concluded that mean temperature (max and min), humidity, and precipitation are changing continuously in an irregular manner in the research area due to abrupt climatic changes occurring spontaneously in AJK. These factors are also found to be dynamic in other developing as well as developed countries of the world, resulting in severe impacts to the agriculture of the globe [27,28]. It was discovered that each variety trial of the wheat showed significant difference in phenological events attributed to changing climate in respected areas and the same has coincided with previous results of the agronomists [29]. It was found that numerical values of four GMC models showed total difference with an average temperature rise of 1.41 °C, 0.88 mm/day changes in precipitation, and about 0.78% change/decrease for humidity under RCP 8.5 with respect to wheat growing season (October–April), as stated by past researchers as well [30,31]. It was noticed that temperature increased more (0.7–1.5 °C) compared to other climatic parameters. The studies depicted that climatic parameters have stronger influence on both wheat flourishment and developmental stages of the wheat and its yield was drastically reduced, which also has meager impacts on life sustenance of mankind [13,32]. DSSAT model was employed to determine, with increased temperature in season, what will be the effect on the grain-filling period, i.e., increased maturity/flowering period or vice versa. For inducing a higher temperature, the stressed function of the model was marked to “ON” to evaluate the wheat yield response towards higher temperature, which proved that with rise of temperature of the day, the yield of the cereal crop (wheat) was significantly reduced. This outcome coincides with previous work whereby an increase in the temperature caused slight shifting of the flowering timing of the plants and similar results were deduced for the wheat yield studies [15,17,33]. The GMC model was employed and it was found that around 72% of variation was measured in the phenology and yield of the two wheat varieties. RMSE for simulated flowering and maturity dates were recorded after 2 and 3 days, respectively, with an average error of 4% for the flowering date and 3% for the maturity date. The RMSE of simulated maize yields was 0.5–0.6 t ha^−1^, with errors generally less than 10%. The study proved that there was a general shift of different morphological and physiological periods of the wheat plants and hence, the change in biophysiochemcial cycle determinates 1 [34,35]. A negative shifting of PDFs was observed for yield from both varieties and the negative tail broadens from Sarala to Manana. In general, the mean projected decreases in wheat yield ranged from 6% at Manana to 11 % at Sarala. Higher probability with respect to yield changes are observed at almost all the sites with 30 % to 0 % interval. Conversely, probability results for changes in yield ranged from −45% to −25% and −25% to 0% intervals with approximately the same results observed at all sites. These results were similar with previous works of the researchers [36]. The experimental trials proved that a projected weight of wheat grain increased at all sites from Chamb to Manana with 6 to 12%, with a probability level of 74% (Chamb) ~ 87% (Manana) during 2015–2100; meanwhile, a decrease in grain number was recorded with significantly larger percentages for historical values of 12% (Chamb) ~ 22% (Manana). CDFs points feasibly decreased for wheat grain number, ranging from 84% at Chamb to 95% at Manana during 2015–2100. So, the greater contribution in wheat yield was attributed to the reduction in grain number due to supraoptimal temperature shifts during flowering period.

This proves that there is a direct correlation of yield with wheat variety, altitude of fields, humidity in air, water table, and general pest or insect infestation on the crop. These findings are correlated with past works of researchers who also stated that there is a strong link between yield of cereal crops and temperature, rainfall, and other altitude parameters [3,14,37]. It was analyzed that wheat varieties that were more resistant to heat or pest infection showed yield and morphological growth in different experimental trial plots. The study proved that with the temperature rise, there is a need to develop and cultivate heat- or temperature-resistant varieties that can grow, flourish, and give maximum yield in harsh and tough environments to cope with the necessities of the human being [5,38]. Therefore, the potential varietal characteristics mentioned above could be a promising adaptation strategy to avoid food crisis under global warming. It was also narrated that use of different fertilizers in a single or synergistic form may alleviate or mitigate yield stress caused due to CC or other environmental factors [39]. It is urged to handle the climate changes on a two-edged-knife model: one is to try “to mitigate drastic climatic changes” and the other or second one is “to develop or prepare adaptation strategy” for producing such cereal crops, which need less water and can grow at elevated temperature with provision of maximum yield and phenetic biomass. The research provides the past analysis of the wheat production record trend and future perspectives of the wheat crop production in the coming fifty years. This model will assist in the provision of such circumstances that can produce maximum yield of wheat for local and national necessities of the food culminating, ultimately, global food security and sustainability.

## 4. Materials and Methods

The present study was designed to evaluate the ongoing climatic changes on heat-resistant wheat varieties in District Bhimber of AJK, Pakistan. In respective aspects, two quantitative approaches were used with two different wheat heat-resistant varieties. The field survey involved growing the selected wheat varieties (Punjab 2018) and (Sialkot–2008) in the field under the ongoing climatic scenario, and phenological changes in the crops’ growing period were noticed in accordance with changing climatic (CC) conditions. Phenological changes included sowing dates, seedling formation, spikelet’s formation, maturity days, and yield. Concentrated information were statistically analyzed and compared with the data obtained from climatic model GCMs. For field survey, commonly used heat-resistant wheat varieties from the study area were selected and plotted in same sq. m. area for two consecutive years (2019–2020 and 2020–2021) at five sites (Chamb, Dander, Sarla, Kalash, and Manana). Four GMCs models were used (CSIRO-Mk3.6.0, GISS-E2-R, IPSL-CM5a-MR, MIROC5, and CCSM4). Respected GMCs were selected as they process historical data values for the selected climatic parameters (temperature and precipitation) with similarities in magnitude and seasonal cycle. These scenarios were simulated for two different time series, i.e., (a) a historic data simulation ranging from 1970–2010 and (b) a higher CO_2_ emission RCP 8.5 scenario [21,22,23,39]. Uncertainties in GCM models regarding temperature and precipitation were biased by using multiple GMCs models. Temperature (maximum and minimum), humidity, precipitation, solar radiation, and soil moisture content (at 25 cm) were bias-corrected and downscaled to 0.5° longitude/latitude via grid quantile mapping from historical climatic data (1970–2010) [40]. Further, R-studio with Q-map package was used for bias-correction using quantile mapping robust empirical quantiles [25,39]. Extrapolation beyond the range of the historical distribution was conducted using a linear interpolation scheme [40,41].

## 5. Experimental Plot

Commonly used wheat varieties in study area were grown at five different sites (Chamb, Dander, Sarla, Kalash, and Manana) under the ongoing climatic scenario. Salient features of sampling plot and climatic conditions are described in Table 1. Plants highly depend on their external environment for formation of food (via photosynthesis) and for exchange of gases. Any change in the external environment has a great influence on the phenology of plants. As with external environmental conditions, soil composition also plays a crucial role in plants’ flourishment. Different soil parameters were studied to evaluate the impact of changing climate on soil and wheat growth and seed development. For analyzing soil moisture content, a SMC dry air gravimeter was used [27,42]; for soil texture, Robinson’s pipette method was used [29]. A core sampler was used for determination of soil bulk density (BD) [29,43]. Available nitrogen was estimated by alkaline permanganate method [31,44]. Olsen’s method for determination of available phosphorus (P) [32,33] was used in the proper way; Potassium (K) was determined through Flame photometry [45]. Soil and water suspension of (1:1) was used for estimation of soil pH through pH meter [34,45]. For analysis of soil organic matter (SOM), the acid digestion method was utilized [34,46].

Moreover, five experimental sites (Chamb), Dander, Sarla, Kalash, and Manana) were selected from District Bhimber as major wheat producers of the areas of two drought wheat varieties, for which there exist high-quality data, i.e., Punjab–2018 and Sialkot–2008. Data were accumulated from regional test performed in District Bhimber AJK, Pakistan from 2019–2021.

## 6. Conclusions

The current study provides past production models of different areas of District Bhimber of AJK, Pakistan and predicts the future trend of food security for the wheat crop. The research proved that climate changes are drastically influencing the wheat biomass and yield of both experimented varieties (Sialkot–2008 and Punjab–2018), but the former one is better than latter one in terms of yield. The outcome or take-home message for the researchers and stakeholder or farmers is that “to adopt two-edged knife (TEK) model for coping the drastic impacts of the climate changes through mitigation and adaptation strategy policy or models”. The use of new varieties (Sialkot–2008 and Punjab–2018) or others that developed drought resistance of wheat is the best method to keep the standard means to cope with the daily necessities of food at the indigenous as well as national level, which will ultimately also supplement the global food works to make food available to each human being. A total twenty different wheat varieties are grown in several rural areas of District Bhimber AJK and, out of these, Sialkot–2008 and Punjab–2018 are prevalently cultivated and results of the present study explain that the wheat varieties “Punjab–2018” and “Sialkot–2008” used in this study are comparatively better in terms of climate resilience. However, further exploration is required in terms of enhancing time duration for data acquisition to monitor climate change impacts.

## Figures and Tables

**Table 1:** Soil structure and climatic parameters observed from fields from District Bhimber AJK, Pakistan during experimental trail.

Years	Soil Type	Soil pH	Total Organic Carbon (%)	P-Content of Soil (mg/L)	Total Saturation Point (%)	Organic Matter Content (%)
2019–2020	Loamy	7.56	10	11	28	0.45
2020–2021	Loamy	7.89	06	08	33	0.31
**Climatic Parameters**						
**Years**	**Max. Temp (°C)**	**Min. Temp (°C)**	**Rainfall (mm)**	**Humidity (%)**	**Soil Moisture Content (%)**	**Solar Radiation** **(kWh/m^2^)**
2019–2020	33	21	0.6 mm	33.5	25	5–7 kWh/m^2^
2020–2021	31	17	0.0 mm	16.5	22	5–7 kWh/m^2^

**Table 2:** Phenological data obtained from experimental plot for two wheat varieties from District Bhimber AJK, Pakistan.

Name of Variety Used															
Sialkot–2008								Punjab–2018							
Sr.No	Area	Year	Flag Leave Formation	Sowing Days	Spikelet’s Formation	Maturity Days	Yield (t/ha)	Sr.No	Area	Year	Flag Leave Formation	Sowing Days	Spikelet’s Formation	Maturity Days	Yield (t/ha)
1.	North Iftikharabad (Chamb),	2019–2020	20	114	163	168	10.4	1.	North Iftikharabad (Chamb)	2019–2020	21	118	167	176	11.5
		2020–2021	21	116	165	170	9.9			2020–2021	22	119	169	173	11.4
2.	Dander kot	2019–2020	20	113	149	171	10.2	2.	Dander kot	2019–2020	21	117	155	176	11.3
		2020–2021	18	115	153	173	10.1			2020–2021	22	114	154	175	11.7
3.	Sarla	2019–2020	21	116	162	169	8.7	3.	Sarla	2019–2020	23	116	164	180	9.7
		2020–2021	17	119	165	174	8.3			2020–2021	20	121	168	176	9.3
4.	Kalash	2019–2020	20	118	169	170	7.7	4.	Kalash	2019–2020	21	119	167	178	8.5
		2020–2021	18	120	170	173	7.1			2020–2021	22	122	172	177	8.4
5.	Manana	2019–2020	21	115	165	172	6.8	5.	Manana	2019–2020	16	115	160	165	5.8
		2020–2021	19	118	168	175	6.4			2020–2021	21	122	169	181	7.5
6.	Mean/stand.dev.	2019–2021	1.4 ± 0.2	1.5 ± 0.3	1.5 ± 0.3	1.2 ± 0.1	1.1 ± 0.1	6.	Mean/stand.dev.	2019–2021	1.7 ± 0.7	1.9 ± 0.6	1.3 ± 0.6	1.9 ± 0.8	1.3 ± 0.8

**Table 3:** Statistical results for phenological data of selected wheat varieties from experimental plots simulated against climatic factors observed from District Bhimber AJK, Pakistan.

Parameters	Sialkot–2008 (V-1)			Punjab–2018 (V-2)		
	Per Day Difference Value	*t*-Test	*p*-Value	Per Day Difference Value	*t*-Test	*p*-Value
Flag leaves formation	9	0.02	0.20	9	0.03	0.36
Sowing days	12	0.01	0.76	12	0.05	0.96
Spikelet’s formation	13	0.2	0.79	13	0.05	0.99
Maturity days	15	0.02	0.65	15	0.03	0.76
Yield	2	0.03	0.78	2	0.04	0.99

**Table 4:** Average monthly changes in temp (°C), precipitation (mm/day), and humidity (%) predicted from four GMC models with a baseline range of 1970–2010 and future range of 2019–2068.

Months	October	November	December	January	February	March	April
CSIRO-Mk3.6.0							
Temp (°C)	1.6	1.5	0.6	0.7	0.9	1.3	1.4
Precipitation (mm/day)	0.2	1.8	1.4	0.4	0.3	1.1	1.0
Humidity (%)	1.2	1.0	1.5	0.6	0.4	0.2	0.6
GISS-E2-R							
Temp (°C)	1.9	1.1	0.9	0.6	0.2	1.5	2.0
Precipitation (mm/day)	0.3	2.3	1.7	1.5	0.7	1.9	0.9
Humidity (%)	0.9	0.4	0.3	0.5	1.3	1.0	2.9
MIROC5							
Temp (°C)	2.7	2.8	1.7	0.3	0.5	1.3	2.5
Precipitation (mm/day)	0.4	2.0	1.9	1.6	0.9	1.5	0.6
Humidity (%)	0.4	2.1	1.5	1.3	0.9	1.7	0.4
CCSM4							
Temp (°C)	2.4	2.1	1.9	0.5	0.6	0.7	0.4
Precipitation (mm/day)	1.1	1.2	0.9	1.1	0.9	1.4	1.3
Humidity (%)	0.2	0.1	0.2	0.6	0.5	0.8	1.3

## Data Availability

The data presented in this study are available in article.
